# Exercise as Treatment for “Stress-Related” Mental Disorders

**DOI:** 10.2174/1570159X22666230927103308

**Published:** 2023-09-27

**Authors:** Antonia Bendau, Moritz Bruno Petzold, Jan Kaminski, Jens Plag, Andreas Ströhle

**Affiliations:** 1 Department of Psychiatry and Neurosciences, Charité - Universitätsmedizin Berlin, Corporate Member of Freie Universität Berlin and Humboldt Universität zu Berlin, CCM, Charitéplatz 1, 10117, Berlin, Germany;; 2 HMU Health and Medical University Potsdam, Potsdam, Germany;; 3 Department of Psychology, MSB Medical School Berlin, Berlin, Germany;; 4 Oberberg Fachklinik Potsdam, Potsdam, Germany

**Keywords:** Sport, fitness, aerobic training, anaerobic training, distress, mental disorders, mental health

## Abstract

The beneficial impact of physical activity on preventing and treating mental disorders has captured growing (research) interest. This article aims to provide a concise overview of essential evidence regarding the effectiveness and underlying mechanisms of physical activity for individuals with mental disorders clustered as “stress-related” conditions. Empirical findings (*e.g*., longitudinal-prospective studies, interventional randomized-controlled-trials, reviews, meta-analyses) regarding the effects of physical activity in the prevention and treatment of stress-related mental disorders are summarized. Furthermore, potential mechanisms underlying these effects are discussed, and recommendations regarding the use of physical activity are outlined. The majority of studies indicate good efficacy of physical activity in prospectively lowering the risk for the incidence of subsequent stress-related mental disorders as well as in the treatment of manifest disorders. Most evidence targets unipolar depressive disorder and, secondly, anxiety disorders. Research regarding posttraumatic stress disorder, obsessive-compulsive disorders, and somatoform disorders is promising but scarce. Physical activity seems to be useful as a stand-alone-treatment as well as in combination with other psychotherapeutic or pharmacological treatments. Multiple intertwined physiological, psychological, and social mechanisms are assumed to mediate the beneficial effects. Recommendations regarding physical activity can orientate on official guidelines but should consider the individual needs and circumstances of each subject. In summary, physical activity seems to be effective in the prevention and treatment of stress-related mental disorders and, therefore, should be fostered in healthcare-settings. Future studies are needed to clarify partly inconsistent patterns of results and to close research gaps, *e.g*., concerning somatoform disorders.

## 1 INTRODUCTION

Whereas the importance of physical activity for physical health is known for many decades, the relevance of physical activity for mental health, respectively its implementation and effectiveness in the prevention and treatment of mental disorders, was rather underestimated for a long time [1-3]. Today, an increasing body of evidence shows promising results regarding the benefits of physical activity in the prevention and treatment of health issues designated as “stress-related”. Stress-related mental disorders traditionally encompass major depression, anxiety disorders, and posttraumatic stress disorder (PTSD) since there is conclusive evidence not only for the prominent role of psychosocial stress for the development as well as maintenance of these conditions but also for a disorder-related physiological alteration of the hypothalamic-adrenal-pituitary-axis (HPA-axis) activity with elevated or lowered levels of the “stress hormone” cortisol [4-7]. In addition, metanalytic data revealed a significant hypercortisolism in patients with obsessive-compulsive disorder (OCD), and previous trials suspected somatoform disorder or somatization to be associated with altered activity of the HPA-axis compared to healthy controls [8-10].

Mental and biological stress appear to be common pathogenetic factors in these conditions [2]. Given that physical activity likely influences physiological, cognitive, and affective stress-related processes [2], it can be assumed that these different mental disorders share essential features regarding the impacts of physical activity on their physio- and psychopathology.

Thus, the objective of this narrative review is to provide an encompassing overview of the efficacy of physical activity in the prevention and treatment of unipolar depressive disorders, anxiety disorders, PTSD, OCD, and somatoform disorders. Despite the presence of systematic evidence for some of these disorders, particularly for unipolar depression, there is currently no comprehensive summary that integrates these five distinct disorder groups while highlighting their similarities and differences with respect to the effects of physical activity. In this review, firstly, the cross-sectional observational evidence pertaining to the associations between physical activity and the prevalence and severity of these stress-related mental disorders is outlined. Subsequently, a summary of longitudinal-prospective studies that investigate the protective effects of physical activity against the incidence of stress-related mental disorders is provided. Thirdly, empirical findings on the application of physical activity in the treatment of these disorders - *e.g*., in intervention studies - are summarized. Alongside this synopsis of essential research findings on the preventive and therapeutic benefits of physical activity for stress-related mental disorders, the review also outlines conceivable physiological/neurobiological, psychological, and social mechanisms that contribute to these positive effects. Further, recommendations (*e.g*., considering the types of activities, intensity, duration, and context), barriers, and contraindications for the use of physical activity are outlined.

We subsume findings on different forms of physical activity. The term physical activity refers to all bodily movements produced by skeletal muscles that result in energy expenditure [11]. Correspondingly, it encompasses movement during leisure time, mobility, work, and household activities. Exercise, fitness training, and sport are smaller facets of physical activity with additional requirements - *e.g*., the pursuit of specific competitive or health-related outcomes. Most studies examining physical activity in the context of treatment of mental disorders refer to endurance training (*e.g*., ergometer training, jogging, cycling), but also studies targeting strength training (*e.g*., weightlifting), martial arts, mind-body interventions, walking, and other forms of physical activity exist.

## 2 CROSS-SECTIONAL OBSERVATIONAL FINDINGS

The increasing exploration of physical activity as a preventive and therapeutic measure originated from cross-sectional observations that revealed connections between physical activity and the prevalence and severity of various mental disorders [2, 3, 12-15]. Numerous correlative observational studies, reviews, and meta-analyses have examined the association of physical activity with the categorical prevalence and dimensional severity of depressive disorders [2, 3, 12-15]. Mostly independent of age, region, cultural background, and target group (inpatient, outpatient, subclinical, general population), these studies concluded that higher activity levels are associated with lower prevalence and severity of depressive symptoms. Regarding anxiety disorders, similar results are evident: Higher levels of regular physical activity are widely cross-sectionally associated with a lower prevalence of anxiety disorders as well as milder anxiety symptoms in adolescents and adults [16, 17]. Problematically, individuals with stress-related mental disorders are often found to be particularly inactive: Compared to participants with other mental health conditions, adults with anxiety disorders even reported the lowest mean levels of physical activity in a study in the UK [18]. These findings using self-reported measures of physical activity have been complemented by objectively measured activity parameters in some studies - for example, using accelerometers in a large study of 96,706 adults with chronic conditions [18]. Likewise, individuals with PTSD are, on average, less physically active than people without PTSD, and the severity of psychopathological PTSD symptoms (*e.g*., anxiety symptoms, depressive symptoms, and avoidance behavior) seems to be inversely correlated with physical activity [19-23]. The prevalence of OCD was also related to low physical activity levels [24]. Similarly, somatoform disorders are often associated with reduced physical activity due to avoidance behavior [25]. In addition, regular physical activity is related to fewer pain symptoms and a reduced presence of symptoms of somatization disorder [26]. Lastly, lower levels of physical activity are also associated with a significantly increased risk of somatization symptoms as well as anxiety and depressive symptoms in the context of burdensome situations (*e.g*., the COVID-19 pandemic) [27-30].

To sum up, physical activity seems to be negatively associated with the presence of stress-related mental disorders and is particularly connected to symptom severity [3]. This hints at a relevant role of physical activity; however, the methodological limitations of cross-sectional observational designs need consideration, as they do not allow any causal conclusions. Thus, it is not possible to conclude if more physical activity lowers psychopathological symptoms or less psychopathological symptoms result in more physical activity or to what extent both pathways co-exist. Further, the influence of unconsidered confounders (*e.g*., socioeconomic variables, physiological factors, psychological variables, *etc*.) might modify associations between physical activity and mental disorders.

## 3 PROTECTIVE EFFECTS OF PHYSICAL ACTIVITY

**Fig. (1):**
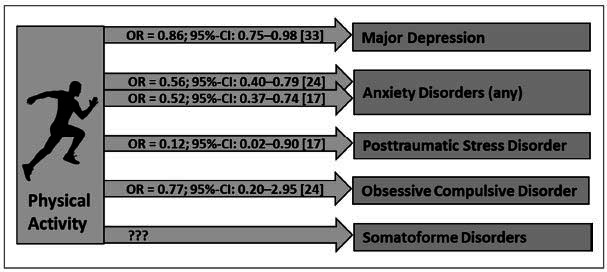
Prospective protective effect of physical activity at baseline regarding the development of “stress related” mental disorders in the subsequent years.

Whereas a large body of cross-sectional evidence demonstrates decreased levels of physical activity in individuals with (compared to individuals without) stress-related mental disorders [2, 3, 15, 31, 32], the protective effect of physical activity on subsequently evolving stress-related mental disorders yet was less intensely studied (Fig. **1**).

Nevertheless, the most evidence regarding the prospective-protective effects of physical activity refers to unipolar depressive disorder [2, 15]. Physical activity prospectively predicted a significantly reduced risk for the incidence of unipolar depressive disorders in the subsequent years in various longitudinal cohort studies [2, 15, 33, 34]. In a large meta-analysis of Schuch *et al*. [33] across 49 unique prospective cohort studies, a total of 266,939 individuals were followed up for 1,837,794 person-years (one up to 26 years after baseline). Compared to individuals with low levels of physical activity at baseline, those with high levels showed lower odds for the incidence of a positive screening for depressive symptoms (adjusted odds ratio (OR) = 0.84; 95%-Confidence interval (95%-CI): 0.79-0.89), respectively a major depression diagnosis (OR = 0.86; 95%-CI: 0.75-0.98) in the subsequent years [33]. The overall effect across all depressive outcome measures was estimated at an OR of 0.83 (95%-CI: 0.79-0.88), with a relatively stable effect across age groups and regions [33]. Even relatively low-intense as well as short activities seemed to be effective compared to physical inactivity [15, 35, 36], and the risk reduction through physical activity was particularly evident in individuals with a pronounced genetic vulnerability [37]. Emphasizing bi-directional relationships, depression at baseline also predicted a reduction in physical activity in the following years in several studies [2, 3, 38, 39]. This should be considered especially concerning comorbid diseases, which are favored by inactivity, as well as potential barriers to the promotion of physical activity in this target group [3].

Regarding the systematic analysis of the potentially protective effects of physical activity for anxiety disorders, only a few prospective longitudinal studies exist [31, 40]. Except for sporadically non-significant effects [16], higher levels of physical activity at baseline were significantly associated with reduced odds of developing an anxiety disorder in the subsequent years [31, 41]. For example, in a general representative population sample of 7,076 Dutch adults, those who regularly engaged in physical activity (60-300 minutes per week) had a 44% reduced risk (OR = 0.56; 95%-CI: 0.40-0.79) for an incident anxiety disorder in the subsequent three years compared to non-active individuals [24]. A risk reduction was evident for all five differentiated types of anxiety disorders (OR: social phobia: 0.35 (95%-CI: 0.18-0.67); generalized anxiety disorder: 0.44 (95%-CI: 0.24-0.79); agoraphobia: 0.45 (95%-CI: 0.25-0.80); panic disorder: 0.46 (95%-CI: 0.25-0.84); specific phobia: 0.52 (95%-CI: 0.36-0.77)) [24]. Similar findings emerged from a prospective longitudinal study of 2,548 adolescents and young adults in Germany [17]: Overall, regularly physically active participants exhibited a 48% reduced risk (OR = 0.52; 95%-CI: 0.37-0.74) of a new onset of any anxiety disorder four years later compared to those with low activity levels (OR: agoraphobia: 0.33 (95%-CI: 0.17-0.77); social phobia: 0.50 (95%-CI: 0.33-1.03); generalized anxiety disorder: 0.51 (95%-CI: 0.14-1.93); specific phobia: 0.60 (95%-CI: 0.40-0.89); panic disorder: 0.68 (95%-CI: 0.26-1.77)). In summary, these longitudinal findings hint at a protective function of physical activity regarding the manifestation of anxiety disorders [3, 24, 31]. Anyhow, the heterogeneity of the designs and results of the underlying studies and possible methodological shortcomings need consideration when interpreting these findings [3, 31, 40, 42, 43]. The promising but still limited evidence of the protective efficacy of physical activity regarding anxiety disorders requires further validation and consolidation through representative longitudinal and, if possible, randomized-controlled studies.

While the research on the preventive role of physical activity concerning PTSD is very limited, the existing studies exhibit encouraging findings [3]. In prospective analyses, the probability of developing PTSD in the subsequent four years was significantly lower in irregularly physically active individuals than in physically inactive individuals (OR = 0.12; 95%-CI: 0.02-0.90); which presents an even stronger probability difference than between regularly physical active *vs.* non-active participants (OR = 0.45; 95%-CI: 0.18-1.15) [17]. In a meta-analysis of 13 prospective studies, a high level (*vs.* a low level) of self-reported physical activity showed a protective effect against the reoccurrence of PTSD (adjusted OR = 0.57; 95%-CI: 0.39-0.85) [44].

Studies regarding the prevalence and incidence of OCD in relation to physical activity are scarce [3]. However, a large epidemiological study in the Netherlands found evidence that, on average, regular physical activity (≥ 4h/week) is associated with a 14% reduced prevalence of OCD (adjusted OR = 0.86; 95%-CI: 0.33-2.25) and, prospectively, with a 23% reduced probability of a new onset of OCD in the subsequent three years (adjusted OR = 0.77; 95%-CI: 0.20-2.95) [24]. Nevertheless, it should be considered that the wide confidence intervals hint at the heterogeneity of results and the lack of statistical significance of these effects.

To date, the protective potential of physical activity regarding the prevention of somatoform disorders was not systematically investigated [3]. Nevertheless, due to overlaps of somatoform symptomatology with other stress-related mental disorders, it can be assumed that physical activity is beneficial in lowering the risk for the incidence of somatoform disorders. Future studies may close this lack of research.

## 4 PHYSICAL ACTIVITY IN THE TREATMENT OF STRESS-RELATED DISODERS

### 4.1 Physical Activity in the Treatment of Unipolar Depressive Disorders

The strive of using physical activity in the treatment of unipolar depressive disorders has been pursued in numerous randomized-controlled trials since the 1970s and has been summarized in more than 20 meta-analyses and systematic reviews, most of which have yielded positive results [2, 12, 15, 45-48]. Recent meta-analyses have further strengthened the evidence that physical activity can reduce depressive symptoms [2, 3, 15, 49]. For example, in a meta-analysis of high-quality studies, physical activity acquired a significant mean effect size (standardized mean difference, SMD) of 0.88 (95%-CI: 0.22-1.54) regarding the reduction of psychopathological symptoms in individuals with unipolar depression (Table **1**) [45]. Overall, physical activity achieved, on average, a medium to large effect on depressive disorders in different meta-analyses [15, 45]. However, the effect sizes of the underlying studies were very heterogeneous and varied from small and insignificant to very large and significant effects [2, 45] - particularly explainable by differences regarding the study design (*e.g*., operationalization of control conditions), samples, diagnoses, assessment instruments, the intervention (type of activity, intensity, duration, frequency, *etc*.) and the heterogeneity of unipolar depressive disorders [2, 3, 15, 45, 50, 51].

A large number of studies investigated the efficacy of exercise-based interventions as stand-alone treatments [2, 15]. The reduction of depressive symptoms through physical activity (*e.g*., 16 weeks of aerobic training) seems even to be comparable to the efficacy of pharmacotherapy and psychotherapy in some studies [2, 3, 15, 52], whereas other findings indicate superior effectiveness of standard treatment procedures while still exhibiting relevant effects of physical activity [2]. In addition to the effectiveness of physical activity as a stand-alone intervention, physical activity was also used to complement or enhance the effectiveness of other treatment interventions (*e.g*., psychotherapy and pharmacotherapy) [3, 53]. For example, a 12-week exercise program in combination with standard pharmacological treatment succeeded in incrementally increasing remission rates in individuals with unipolar depression [54]. Also, the combination of psychotherapy and physical activity showed greater effects than each treatment method alone [55]. Most studies focus on major depressive disorder, but evidence examining other forms of unipolar depressive disorders grows, including postpartum depressive disorder and seasonal affective disorder [3]. Based on the promising level of evidence, treatment guidelines included the recommendation of physical activity as a supplementary measure in the treatment of unipolar depressive disorders [56].

The majority of interventions regarding the anti-depressive effects of physical activity refer to guided aerobic training programs with eight to 20 weeks duration and focus on medium- and long-term effects [2, 15]. However, there also exists evidence for acute efficacy, respectively, immediate mood-lifting effects of single exercise sessions [57]. Furthermore, an increasingly broad variety of forms of physical activity (*e.g*., strength training, yoga, martial arts, *etc*.) is investigated in the context of the treatment of depressive disorders. Besides enhancing activity, particularly cutting mentally passive and sedentary inactivity (*e.g*., watching television on the couch) seems to be relevant to ameliorate depressive symptoms [34].

### 4.2 Physical Activity in the Treatment of Anxiety Disorders

Compared to the extensive research on the therapeutic effects of physical activity in the treatment of unipolar depressive disorders, evidence regarding anxiety disorders is still sparse [2, 31, 58]. Nevertheless, several studies indicate the efficacy of physical activity in the acute as well as long-term treatment of anxiety disorders [31].

For example, in a randomized-controlled cross-over design, a single 30-minute moderately intense aerobic treadmill session significantly (compared to a low-intensity control condition) reduced anxiety levels during a subsequent dental prophylaxis treatment in people diagnosed with dental phobia [59]. For panic disorder, promising findings were evident as well. A moderately intense aerobic training session succeeded in reducing the intensity as well as the frequency of subsequent panic attacks induced by medication [60] and CO_2_ [61]. In summary, these findings suggest that single training sessions, especially immediately before exposure to anxiety-provoking stimuli, can alleviate acute anxiety symptoms in individuals with anxiety disorders [31].

Besides these acute anxiolytic and anti-panic effects, several studies aimed to investigate medium- and long-term anxiety-reducing effects of physical activity in individuals diagnosed with anxiety disorders: A meta-analysis across six randomized-controlled trials (with a total of 262 adults) found, on average, a medium-size effect of aerobic training on symptom reduction (SMD = -0.58; 95%-CI: -1.0, -0.76) [62]. In a systematic review of 12 randomized-controlled trials, physical activity interventions even achieved an effect size comparable to pharmacotherapy and/or psychotherapy [63]. Other studies indicated less but still good efficacy of aerobic training compared to cognitive behavioral therapy [43, 64] and pharmacological treatment [43]. Further, the positive effects of physical activity are enhanced by the absence of negative side effects [3, 43, 65]. The promising findings from randomized-controlled trials are supported by a prospective longitudinal study: The likelihood of remission of a diagnosed anxiety disorder in the subsequent three years was overall increased by 58% (OR = 1.58; 95%-CI: 1.09-2.29) in regularly physically active compared to less active individuals [24]. For specific phobia (OR = 1.58; 95%-CI: 0.09-2.65) and social phobia (OR = 1.57; 95%-CI: 0.77-3.21), the mean effect was the strongest (panic disorder: OR = 1.38 (95%-CI: 0.48-3.98); agoraphobia: OR = 1.16 (95%-CI: 0.29-4.61); generalized anxiety disorder: group too small to calculate a meaningful OR) [24]. While predominantly aerobic forms of exercise, such as jogging or ergometer cycling, have been considered, studies with smaller samples hint at the good efficacy of high-intensity interval training in individuals with panic disorder and generalized anxiety disorder [66, 67]. Other forms of physical activity, such as martial arts, yoga, and tai chi, have also been shown to yield positive anxiolytic effects in several studies [31, 32].

Besides the use of physical activity interventions as stand-alone treatments, physical activity can potentially be applied as an augmentation to enhance the effectiveness of other treatment modalities [3, 31, 53]. For example, there is preliminary evidence that exercising immediately before an exposure session (in the context of cognitive behavioral therapy) may increase the efficacy of the exposure therapy respectively, the corrective learning experience [3, 68-70]. This can be partially attributed to the beneficial effects of physical activity on neuroplasticity processes, such as the stimulation of brain growth factors, which are critical for the consolidation of adaptive changes in fear memory structures during extinction [3, 68-71]. Besides acutely applied physical activity for augmentation, aerobic exercise programs over several weeks also appear to tendentially increase the effectiveness of cognitive behavioral therapy in the longer run, *e.g*., regarding the treatment of diagnosed panic disorders [70]. In official national [71] and international [72] treatment guidelines, physical activity (aerobic training) is advised as a complementing measure for treating anxiety disorders.

### 4.3 Physical Activity in the Treatment of PTSD

While numerous studies demonstrate the positive effects of physical activity on various symptoms associated with PTSD, such as sleep disturbances, cognitive dysfunction, anxiety, and depressive symptoms, few studies specifically address the core symptoms of people with PTSD [2, 3, 22, 73]. According to a review of three studies, five- to ten-week aerobic training programs achieved acute improvements in PTSD severity, anxiety, and depressive symptoms in children, adolescents, and young adults with PTSD [74]. These positive effects persevered at the follow-up one month later [74]. In adults, similarly promising results were evident: A meta-analysis of four studies involving 200 adults and various forms of interventions (yoga, aerobic, and strength training; six- to 12-week programs) showed reductions in psychopathological symptoms [75]. In addition, physical activity may provide augmentative effects in combination with other treatments: In a small randomized-controlled study with nine young adults with PTSD, 30 minutes of treadmill training before trauma exposure significantly increased the therapeutic effect of prolonged exposure (compared with exposure without prior physical activity) [76]. Another randomized-controlled study with 81 adults diagnosed with PTSD evaluated the efficacy of a 12-week aerobic training program in combination with the standard treatment (psychotherapy, pharmacotherapy if necessary, and group therapy) [23]. Beyond the effects of the standard treatment, aerobic training contributed to significant improvements in PTSD severity, as well as decreases in anxiety, depressive symptoms, stress, and sleep disturbances and favorable changes in metabolic parameters and cardiovascular health [23]. A qualitative study with traumatized refugees complements these quantitative findings by showing significant improvements in physical and mental health through physical activity in a group setting as a component of the overall treatment [77]. Specifically, participants reported enhancement of self-confidence and self-awareness, relaxation through distraction and intermission of the confrontation with daily stressors, strengthening of social interactions, and decreases of social fears and isolation due to the group exercise [77]. Divergently, in some other studies, the findings were partially inconsistent [3]. For example, in a study with traumatized adult refugees in Denmark, the combination of physical activity and standard treatment did not add significant improvements compared to standard treatment alone [78]. Further, heterogeneous and small samples and the partial lack of appropriate control conditions limit the generalizability of the results regarding the effectiveness of physical activity on PTSD [74, 75].

Nevertheless, the potential of physical activity in the treatment of PTSD is also reflected in the fact that endurance training is explicitly recommended as an adjuvant intervention method in current national [79] and international [72] treatment guidelines for the treatment of PTSD. The possible indications hereby seem to range from subsyndromal symptoms to therapy-resistant or even complex PTSD [3, 22, 80, 81].

### 4.4 Physical Activity in the Treatment of Obsessive-compulsive Disorders

To date, there are very few studies on the possible application and effectiveness of physical activity in the treatment of OCD. Since the symptoms of OCD overlap to a certain extent with the symptoms of anxiety disorders, many findings regarding anxiety disorders can presumably also be applied to OCD [41].

In a correlative longitudinal study, regular physical activity was strongly associated with a higher likelihood of remission of a pre-existing OCD in the subsequent three years (adjusted OR = 2.65; 95%-CI: 0.14-4.36) [24]. This may indicate a positive influence of physical activity on the progress of OCD, but due to the very small sample size as well as the correlational design, no causal conclusions are possible [3, 24, 82]. At least some experimental designs have attempted to lay a fundament of evidence for the efficacy of physical activity in the treatment of OCD [3, 83]. For example, a 12-week aerobic exercise program, compared with a psychoeducational control condition, achieved significant improvements in mood and amelioration of anxiety symptoms and obsessive-compulsive behaviors in individuals with treatment-resistant OCD [83]. The efficacy of a 12-week aerobic training program in combination with the standard treatment (pharmacotherapy or cognitive-behavioral therapy) on reducing the severity of OCD accomplished a large effect (effect size: Cohen’s d = 1.69) [84]. For half of the participants, clinically significant symptom reduction even persisted at the follow-up six months later [84]. Similar positive results were also seen in a study examining the efficacy of an aerobic “home workout” as an augmentative adjunct to standard treatment [82]. However, it is important to note that these findings are only preliminary indications that need to be confirmed reliably in further randomized-controlled trials with appropriate control conditions and larger sample sizes [3].

### 4.5 Physical Activity in the Treatment of Somatoform Disorders

To date, no randomized-controlled trials are investigating the efficacy of physical activity in the treatment of somatoform disorders [3]. However, preliminary findings concerning symptoms of somatization, anxiety, and depression in the context of other disorders provide initial evidence for the potential benefits of physical activity on somatoform disorders [26-29, 85, 86]. After one week of increased daily physical activity, individuals with the unipolar depressive disorder as well as individuals with somatoform disorders showed significant reductions in somatoform symptoms [85, 86]. The symptom improvements by gradually intensified short-term physical training were, however, not systematically associated with biological changes (serotonergic or inflammatory parameters) in individuals with multiple somatoform symptoms [3, 86].

In contrast to the currently very scarce empirical research in this regard, physical activity is already officially recommended in guidelines for the treatment of somatoform disorders [3, 25, 87]. Graded physical activation, neither overloading nor excessive resting, is advised as a key therapeutic measure [87]. Physical activation should best begin in the form of small behavioral changes that are gradually increased in an individualized manner [25, 87]. In particular, pleasurable exercise, regardless of its form and its integration into everyday life, has proven effective. Group exercise activities can also achieve positive effects through social interaction in individuals with somatoform disorders.

## 5 MECHANISMS OF THE EFFECTS OF PHYSICAL ACTIVITY

### 5.1 Physiological/Neurobiological Mechanisms

**Fig. (2):**
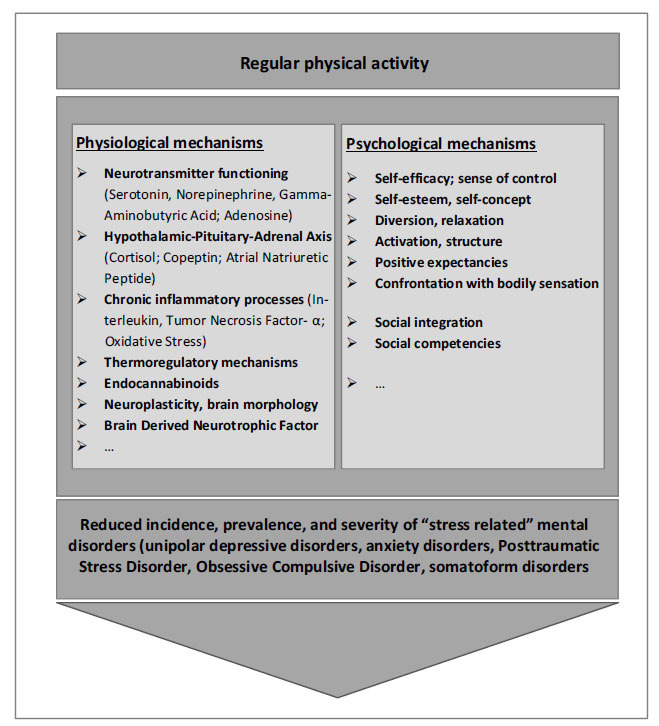
Potential mechanisms mediating the positive effects of regular physical activity on the incidence, prevalence, and severity of “stress-related” mental disorders.

A variety of physiological mechanisms is assumed to be involved in mediating the beneficial effects of physical activity on stress-related disorders [3, 63]. In the following, an overview of possible physiological/neurobiological mechanisms is presented without claiming to be exhaustive (Fig. **2**). Moreover, it is important to be aware of the lack of clear, high-quality evidence concerning these mechanisms in humans with stress-related disorders. Studies partially exhibited contradictory results, and regarding some of the mechanisms, *in-vivo* studies with humans are not possible and empirical conclusions only rely on animal studies. Further, it may be hardly possible to examine the detangled “pure” effect of single mechanisms because they are presumably strongly intertwined.

#### 5.1.1 Neurotransmitter Functioning

Adaptive changes in neurotransmitter systems are assumed to play an important role in the symptom-reducing effects of physical activity in stress-related disorders [2, 3, 31, 51]. The neurotransmitter Serotonin (5-hydroxytryptamine, 5-HT) exerts a relevant influence on the mood and behavior of humans, and distortions in the serotonin system are associated with various symptoms [3, 31, 41, 51, 88]. While physical activity is usually associated with an acute increase in serotonin activity directly after the exercise [89], it is assumed (primarily based on the evidence of rodent studies) that repeated stimulation of serotonin activity by regular physical exercise may lead to an adaptive regulation of serotonin receptors [2, 51, 88, 90]. These long-term regulatory changes in the serotonin system through regular physical activity were, in turn, associated with lower depressive symptoms and less anxiety-related behaviors, such as more pronounced social exploration behavior, in animal studies [88]. First studies with participants without current mental disorders support the assumption of an adaptive effect of physical activity on serotonergic functions, whereas, in a review by Schuch *et al.* [91], overall, neither significant acute nor long-term effects of physical activity on precursors (*e.g*., tryptophan), metabolites, and other index biomarkers (*e.g*., prolactin) of serotonergic functions were found in individuals with depression. Another neurotransmitter that is assumed to be relevant for the beneficial effects of physical activity is norepinephrine [15]. Norepinephrine exhibits a complex multilayered interplay with depressive, anxiety, and other stress-related symptoms [3, 15, 92, 93]. For example, norepinephrine appears to be involved in the development of panic disorders and can both increase and decrease anxiety symptoms [3, 92]. In studies with rodents, regular physical activity led to a reduction in anxiety-related behaviors mediated by inhibition of norepinephrine activity [92, 94]; a similar mechanism could potentially be involved in the effects of physical activity in humans. Furthermore, the changes in serotonergic and noradrenergic systems (that were examined in animal studies) induced by regular physical activity are similar to the mechanisms assumed to be central to the transport effects of psychotropic drugs in the treatment of depression and anxiety disorders [3, 93]. In addition, gamma-aminobutyric acid (GABA), an amino acid that acts as an inhibitory neurotransmitter in the central nervous system, is also discussed as a mediator of physical activity [3, 95, 96]. Considering the anxiolytic activity of benzodiazepines which potentiate the effect of GABA, it has been suggested that GABA-related changes induced by physical activity may partially mediate the effects of physical activity in individuals with stress-related disorders. Findings from animal studies (that have not yet been undoubtedly transferred to human individuals) indicate that regular physical activity leads to long-term downregulation of GABA_A_ receptors, thereby increasing the efficacy of GABA and resulting in reduced anxiety symptoms [96]. This is further supported by a randomized-controlled trial with a 12-week yoga intervention that resulted in an acute increase in GABA concentration in the thalamus, which was, in turn, associated with an anxiety-reducing effect [95]. With regard to neurotransmitter functioning, adenosine, an inhibitory neuromodulator that affects synaptic dopamine and glutamate transmission, is also discussed to be relevant for mediating effects of physical activity since animal studies have shown effects of physical activity on adenosine receptors and associated reductions in anxiety symptoms [15, 93].

#### 5.1.2 The Hypothalamic-pituitary-adrenal Axis (HPA-axis)

The HPA-axis (also known as the “stress hormone system”) and the sympathetic nervous system are often exhibiting regulatory disturbances, predominantly an overactivity when individuals are chronically or high-frequently confronted with psychological or physiological stress - such as in the context of stress-related disorders [3-10, 97-99]. Long-term over-pronounced stress reactions, in turn, lead to several adverse effects. For example, prolonged cortisol exposure yields multiple neurotoxic effects (including desensitization of receptor cells, cell death, and reduced neurogenesis) in prefrontal and hypothalamic brain regions [100]. A meta-analysis subsuming five studies found that physical activity succeeded in reducing cortisol levels in participants with major depression (SMD = -0.65; 95%-CI: -1.30-0.01) [101]. In contrast, Schuch *et al*. [91] found neither acute nor long-term physical-activity-induced changes in cortisol levels in a systematic review of studies regarding unipolar depressive disorders. Nevertheless, concerning copeptin, another biomarker of overall stress levels and activity of the HPA-axis, promising results were evident: A single exercise session caused a moderate increase in copeptin levels, whereas a 12-week endurance program achieved a chronic reduction in copeptin levels in the long run [91]. With regard to the HPA-axis, also the atrial natriuretic peptide (ANP) deserves attention. ANP is a hormone that inhibits HPA-axis-related activity and may have anxiolytic and stress-relieving properties [93, 97, 102]. For example, a 30-minute aerobic training session on a treadmill significantly increased plasma ANP levels while acutely reducing anxiety symptoms in individuals with panic disorder [102]. Hereby, the extent of anxiety reduction was directly related to the increase in ANP levels; consequently, it can be suggested that ANP represents one of the mechanisms mediating the beneficial effects of physical activity on anxiety reduction and maybe for ameliorating other psychopathologies, too [3, 93, 102]. In addition, ANP is considered to be a protective marker against the presence and incidence of cardiovascular diseases and may also represent a mechanism for the bidirectional improvement in cardiovascular health associated with the reduction of psychiatric symptoms [3, 91].

#### 5.1.3 Chronic Inflammatory Processes

Depressive disorders, anxiety disorders, and other stress-related mental disorders are frequently associated with subthreshold inflammatory processes, respectively elevated chronic inflammatory markers (*e.g*., the proinflammatory markers interleukin-1, interleukin-6, and tumor necrosis factor-α) [3, 32, 100]. Directly after physical exercise, such inflammatory processes in the body are enhanced, but in the long term, physical activity is associated with an adaptive decrease in chronic inflammations in individuals with and without mental disorders [100, 103, 104]. This, in turn, potentially leads to an improved mood, less depressive symptomatology, and reduced anxiety. For example, an adaptive change in interleukin-1β induced by regular physical activity was positively associated with a decrease in depressive symptoms in a study on humans with unipolar depression [91, 100]. Further evidence for this potential mechanism is provided by a systematic review of 14 studies that found a significant reduction in depressive symptoms with anti-inflammatory medication [100]. With regard to chronic inflammation, oxidative stress should be considered, too. Oxidative stress refers to the imbalance between oxidative and antioxidative processes caused by free radicals, which seems to be involved in the pathophysiology of various stress-related mental disorders [100, 105]. Regular physical activity seems to reduce oxidative stress and, thereby, decrease the production of pro-inflammatory cytokines [3, 100, 105].

#### 5.1.4 Thermoregulatory Mechanisms

Thermoregulatory mechanisms are relevant for body self-regulation, and regular physical activity seems to adaptively influence these mechanisms in the long run by enhancing their robustness towards arousal and stress [3, 15, 97, 106]. This might contribute to improvements on the mental level as well, yet clear evidence in this regard is lacking.

#### 5.1.5 Endocannabinoids

Endocannabinoids are involved in numerous physiological, immunological, emotional, and cognitive processes and may be influenced beneficially by physical activity [3, 63, 107, 108]. For example, an aerobic exercise program adjunctive to standard treatment succeeded in increasing the concentration of the circulating endocannabinoid anandamide [107], which, in turn, is associated with less anxiety and depressive symptoms [108]. However, further research is needed to clarify these mechanisms.

#### 5.1.6 Endorphins

The assumption that an increase in endorphins (endogenous opioid peptides) causes positive effects of physical activity are widespread, but not yet empirically unambiguously supported [3, 32, 41, 93, 97].

#### 5.1.7 Neuroplasticity, Adult Neurogenesis, Brain Morphology

Stress-related mental disorders are partially associated with brain morphological abnormalities, such as reduced hippocampal volume [3, 15, 51, 109]. In animal studies, physical activity led to increases in regional cerebral blood flow and neurotrophic growth factors, thereby stimulating the formation and connection of neurons (neurogenesis). Thus, an increase in the volume of the hippocampus and the prefrontal and anterior cingulate cortex and improvements in the structure and function of the white and gray matter of the brain were evident [2, 33]. Since these areas are multidimensionally relevant for stress-associated psychopathologies, these neuroplastic processes could be involved in the beneficial effects of physical activity on stress-related disorders. However, some studies contrastingly did not find significant effects of physical activity on brain morphology and it might be that the effect on neuroplasticity tends to be stronger at an earlier age because the capacity for neuroplasticity changes seems to decrease over the lifespan [100].

#### 5.1.8 Neurotrophin Hypothesis/Brain-derived Neurotro-phic Factor (BDNF)

The neurotrophin BDNF is involved in the consolidation of memories, synaptic plasticity, and associative learning, and it is assumed that imbalances in BDNF levels may contribute to the development and maintenance of stress-related disorders; particularly concerning processes of fear learning and fear memory [61, 93, 104, 110]. Individuals with depression as well as people with anxiety disorders and PTSD, exhibit relatively low BDNF levels on average [104, 111]. Enhancement of the BDNF synthesis was significantly stimulated by physical activity in randomized-controlled trials and seemed to influence the acquisition, consolidation, and extinction of fear memory structures acutely *via* key glucocorticoid and noradrenergic systems [93, 111-114]. In the first studies, evidence from animal-based research on acute exercise and fear memory structures has been extended to human populations and succeeded predominantly in replicating the effects of exercise on emotional memories and extinction consolidation [114]. BDNF is accordingly supposed to be involved in the augmentative effect of physical activity on the effectiveness of exposure-based psychotherapy. For example, individuals with panic disorder who had higher levels of BDNF in their blood serum showed a stronger average effect of exposure-based interventions than those with low BDNF concentrations [97, 115].

#### 5.1.9 Sleep Quality

Stress-related disorders are often related to disturbances in falling and staying asleep, nightmares, and numerous other impairments in sleep quality [71, 116]. Physical activity has been shown to significantly improve sleep quality in numerous studies [97]; partially explained by exercise-driven physical processes (*e.g*., an increase in body temperature) that promote sleep and also reduce depressive and anxiety-related symptoms [3, 15, 97]. Physical activity in military veterans with PTSD led to a significant reduction in hyperarousal, especially when the veterans had previously suffered from poor sleep quality; this suggests that physical activity might be able to break the self-reinforcing vicious cycle of sleep complaints and symptom exacerbation in PTSD [80].

#### 5.1.10 Breathing

Breathing is often unconsciously involved in anxiety reactions (*e.g*., panic attacks) in stress-related mental disorders [32]. In addition, breathing can influence processes of bodily relaxation, too. Physical activity (especially activities with a particular focus on breathing) can help to identify, control, and use breathing more consciously and therefore reduce symptom burden.

### 5.2 Psychological and Psychosocial Mechanisms

Multiple psychological and psychosocial processes seem to be involved in transporting the beneficial effects of physical activity; the most commonly assumed mechanisms are presented in the following (Fig. **2**) [3, 63].

#### 5.2.1 Self-efficacy and Sense of Control

People with stress-related disorders often experience little sense of control and have low general as well as domain-specific self-efficacy; in other words, they often feel not able to achieve specific goals and to control events and conditions in their life [3, 15, 31, 53, 93]. This perceived lack of control, low coping ability, and feelings of being a victim of external circumstances are often associated with perceived helplessness and distress. In this regard, physical activity can provide a method to achieve defined goals (*e.g*., to walk five kilometers or swim a specific distance) and thereby gain a sense of control and self-efficacy. Enhanced domain-specific self-efficacy and control experiences may gradually transfer to other areas of life [3, 97, 117].

#### 5.2.2 Self-esteem and Self-concept

The constructs of self-esteem and self-concepts are closely connected to self-efficacy and control beliefs [93, 100]. Individuals with depressive, anxiety, and other stress-related mental disorders often exhibit low self-esteem and rather negative self-concepts [104, 118]. They often tend to perceive themselves as less worthy than others and adversely attribute failure to their selves, whereas success is attributed externally. Physical activity may buffer these maladaptive cognitions and behaviors and stimulates benevolent attribution styles. The findings of randomized-controlled trials imply that higher self-esteem and positive self-concepts can be promoted by regular physical activity [119]. In a meta-analysis of 113 studies, the effect of physical activity on global self-esteem was small but significant (Cohen’s *d* = 0.23), and the largest effect was evident regarding the enhancement of the physical self-concept [104].

#### 5.2.3 Diversion and Relaxation

Physical activity can provide a possibility of relaxation as well as counterbalance and diversion from straining cognitions, perceptions, behaviors, and situations, *e.g*., by breaking negative thought circles and rumination [53]. This, in turn, seems to ameliorate psychopathological symptoms in stress-related mental disorders [93, 97].

#### 5.2.4 Activation and Structure

Individuals with stress-related mental disorders often exhibit high levels of inactivation and a lack of structure in daily life which, in turn, are associated with deteriorated mental well-being [2, 3]. Physical activity, particularly regularly scheduled, provides an anchor for structure and a stimulus for activation.

#### 5.2.5 Positive Expectancy/Placebo Effects

Positive expectations regarding the effects of physical activity can also produce symptom-reducing effects [120]. Randomized-controlled trials aim to mitigate the impact of placebo effects by implementing appropriate control conditions. However, since it is often not feasible to blind participants to their condition, even well-controlled interventions may be susceptible to the influence of placebo effects [3, 120].

#### 5.2.6 Confrontation/Exposure with Bodily Sensations

Exposure to anxiety-provoking stimuli and anxiety-related mental and physical responses represents a core element of cognitive-behavioral treatment for anxiety disorders and PTSD [71]. Physical activity can elicit somatic sensations that feel very similar to those of a physiological fear response [3, 31, 53]. As a result, individuals with anxiety disorders often tend to misinterpret bodily activation induced by physical activity as the beginning of an anxiety reaction and, therefore, avoid and fear physical activity [31, 121]. This phenomenon is called “exercise anxiety”. On the one hand, it partially explains low activity levels in individuals with anxiety disorders and PTSD; on the other hand, it allows the therapeutic confrontation/exposition with feared bodily sensations to achieve habituation to them. This serves the reduction of fear sensitivity and emotional avoidance behavior and fosters a gradual decoupling of learned problematic associations between fear-inducing stimuli and fear reactions.

#### 5.2.7 Social Integration and Competencies

Physical activity can improve social interaction, integration, and competencies in children, adolescents, and adults; this is particularly useful concerning these areas, often being impaired in individuals with depressive, anxiety, and other stress-related mental disorders [100, 122, 123]. To ensure positive social experiences, it is important to take into account the worries and fears of people with stress-related disorders regarding social situations - *e.g*., the fear of performing bad or negative evaluations by others [3, 71].

## 6 RECOMMENDATIONS REGARDING EXERCISE TREATMENT

### 6.1 Type of Activity, Intensity, Duration, and Context

**Fig. (3):**
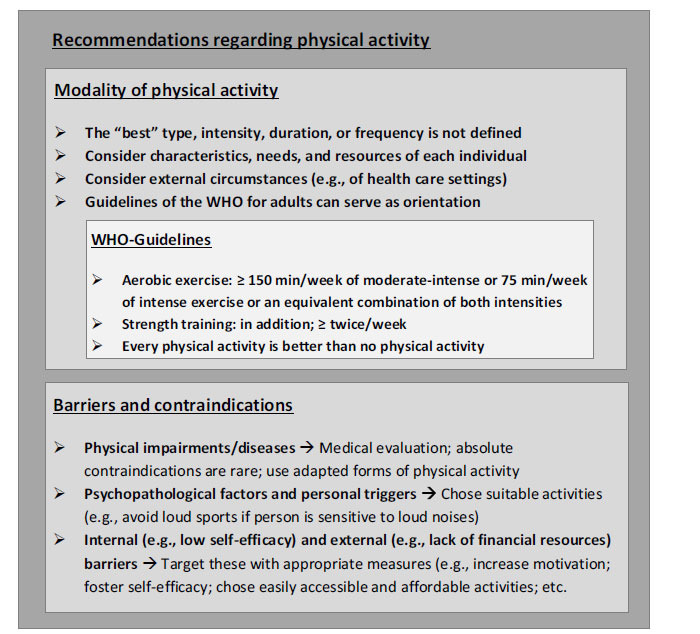
Recommendations for the use of physical activity in the treatment of “stress-related” mental disorders.

It is not possible to define the “best” type, intensity, duration, or frequency of physical activity for the prevention and treatment of stress-related mental disorders [15, 48, 56]. First, because the empirical evidence does not allow clear conclusions in this regard and, second, because each individual has specific characteristics, needs, resources, and external circumstances that influence which activity modalities fit the best (Fig. **3**) [3].

Nevertheless, the guidelines of the World Health Organization (WHO) for the general adult population [124] can serve as a broad orientation, provide the base for most existing official recommendations, and are also listed in treatment guidelines, *e.g*., for anxiety disorders [71]. The WHO recommends a minimum of 150 minutes of moderate-intensity or at least 75 minutes of intense aerobic exercise per week (or an equivalent combination of both intensities) [124]. In addition, it advises adding strength training twice a week. Further, it emphasizes vigorously that every exercise session is helpful [124]. Thus, even a single workout or even a short walk is beneficial for physical and mental health. However, to achieve substantial and long-lasting effects, it is advisable to perform a greater amount of exercise regularly [15, 124]. At least to a certain extent, “the more, the better” seems to be true when it comes to dose-effect-relations. Inordinate training amounts with negative effects (*e.g*., overtraining, sports addiction) are rarely observed and only need consideration at the sight of alarming signs or particular risk constellations [3].

Aerobic training (*e.g*., treadmill jogging, ergometer cycling) has been studied most frequently regarding its efficacy in the treatment of stress-related mental disorders - especially because it is easy to operationalize and standardize [3]. As a result, aerobic activity is anchored most strongly in official recommendations [71, 124]. However, the benefits of strength training have also been proven in multiple studies [3]. Further, positive evidence regarding the efficacy of various other forms of physical activity, such as martial arts, yoga, tai chi, qigong, and dancing, grows [2]. Even completely unstructured forms of physical activity as well as activities in daily life - *e.g*., in the household or garden - seem to be beneficial for physical and mental health [3, 125]. Further, particularly the reduction of sedentary, inactive phases seems to be crucial concerning the prevention and treatment of stress-related mental disorders [34, 56, 71, 124, 126, 127].

Regarding the setting and context of physical activity, there is no definite superiority of any certain modality evident [2]. Indoor as well as outdoor activities proved good efficacy; the same applies to group *vs.* single activities and unguided *vs.* video guided *vs.* personally instructed training [2, 3]. In general, orientation to individual preferences and personal conditions, as well as external circumstances, seems to be useful when choosing types, intensity, duration, and contexts of physical activity [2, 116, 128]. Further, it is crucial to consider the baseline level of activity, knowledge, and experience to foster realistic goals and to increase physical activity gradually to avoid physical overstraining and psychological frustration [2-4, 25, 53].

### 6.2 Barriers and Contraindications

Caution is required when advising physical activity for individuals with (chronic) physical impairments or manifest diseases [129]. Since stress-related mental disorders are often accompanied by an increased risk of physical comorbidities, this is of particular relevance [2, 93, 129]. Nevertheless, absolute contraindications regarding exercise are rare and in most cases, appropriately adapted physical activity is possible and beneficial.

Individuals with stress-related mental disorders are often particularly little active and find it difficult to engage in regular physical activity, which is also reflected in low adherence rates to exercise programs [3, 116, 130, 131]. Thus, motivation and volition should be specifically promoted to foster the initiation and maintenance of an adaptive activity level [3, 53, 132]. Common barriers to physical activity in individuals with stress-related mental disorders are severe psychopathological symptoms, side effects of medication, somatic comorbidities, social isolation, a lack of knowledge or adverse attitudes regarding physical activity, few or negative experiences with physical activity, low self-efficacy, sociodemographic factors, and insufficient financial, structural, and time resources [3, 129, 133]. Such barriers should be identified, considered, and targeted appropriately.

In addition, specific psychopathologies and triggers should be taken into account [3]. For example, noisy or crowded activities might trigger flashbacks and symptom escalation in individuals with PTSD [79]. Confrontation with such stimuli by accident without adaptive therapeutic contextualization should be avoided.

In the context of managing stress-related mental disorders, it is advisable to implement physical activity programs across stationary and ambulatory settings to achieve an adaptive level of activity that endures beyond the cessation of other treatments and extends into personal life [3, 132]. To approach this goal, interprofessional and interdisciplinary collaboration and coordination seem to be required [129, 134-136].

## CONCLUSION

In summary, physical activity seems to be an effective tool in the prevention and treatment of stress-related mental disorders. Especially regarding unipolar depressive disorders, the body of positive evidence is large, followed by studies regarding anxiety disorders. Concerning PTSD, OCD, and somatoform disorders, research investigating the efficacy of physical activity in prevention and treatment is still rare, but the first studies yield promising results. Future studies may elaborate on these effects in more in-depth. Additionally, the heterogeneity in results (*e.g*., the range of the observed effects on stress-related mental disorders from non-significant small to very large effect sizes), study designs, and quality need consideration. Physical activity seems to be useful as a stand-alone treatment, as well as an augmentative measure in combination with other psychotherapeutic or pharmacological treatments. Multiple interrelated physiological, psychological, and social mechanisms are assumed to mediate the beneficial effects of physical activity. Recommendations regarding physical activity can use official guidelines as orientation but should also consider the individual needs and circumstances of each person.

## Figures and Tables

**Table 1:** Evidence level, effect sizes, and sample sizes of studies regarding the exercise treatment on disease severity in adults with stress-related mental disorders.

**Disorder**	**Evidence Level**	**Effect Size**	***N***	**References**
Unipolar depression	1a	*g* = -0.68 SMD = -0.88 *g* = -0.82 *g* = -0.79 *g* = 0.64 *g* = -0.87 to -1.38 (mind-body exercise) g = -0.51 to -1.02 (aerobic exercise) *g* = -0.41 to -0.92 (resistance exercise)	977 267 771 455 1308 596	[137] [45] [48] [46] [49] [138]
Anxiety disorders	1a	*d* = -1.23^a)^ SMD = -0.58 Δ = 0.31 SMD = -0.41	61 262 922 675	[43] [62] [139] [58]
Panic disorder/agoraphobia	1a	*d* = -1.17^a)^	33	[43]
Generalized anxiety disorder	1b	*g* = -0.45^a)^ *d* = 0.71	30 44	[140] [141]
Social phobia	1a	*d* = -0.24^x^	749	[142]
Obsessive-compulsive disorder	4	*d* = -0.17^x^	56	[82]
Post-traumatic stress disorder	1a	*g* = -0.35 SMD = 0.46	200 605	[75] [73]
Somatoform disorder	5	n.a.	-	-

**Note:** a) Pre-Post, x) not significant.

## LIST OF ABBREVIATIONS

ANP: Atrial Natriuretic Peptide
BDNF: Brain-derived Neurotrophic Factor
CI: Confidence Interval
GABA: Gamma-aminobutyric Acid
GAD: Generalized Anxiety Disorder
HPA-axis: Hypothalamic-pituitary-adrenal Axis
OCD: Obsessive-compulsive Disorder
OR: Odds Ratio
PTSD: Posttraumatic Stress Disorder
SMD: Standardized Mean Difference
WHO: World Health Organization
